# Coping Mechanisms of Psychiatric Nurses in Child Mental Health Environments in North West Province, South Africa: A Qualitative Call for Urgency

**DOI:** 10.3390/ijerph20176625

**Published:** 2023-08-22

**Authors:** Rorisang Mary Machailo, Molekodi Jacob Matsipane, Daleen Koen

**Affiliations:** School of Nursing, Faculty of Health Sciences, Mahikeng Campus, North West University, Mahikeng 2745, South Africa; molekodi.matsipane@nwu.ac.za (M.J.M.); daleen.koen@nwu.ac.za (D.K.)

**Keywords:** environment, coping mechanism, children, child mental health, psychiatric nurses

## Abstract

There is currently a growing understanding of child mental health. However, with little attention and investment from decision-makers, the prevalence of child mental health challenges shows no signs of diminishing. Psychiatric nursing is a process in which the major knowledge and skills dealing with the interpersonal and intrapersonal dynamics of human beings are practised. These nurses have to cope with this demand in a scant clinical child psychiatric environment. An exploratory, descriptive, and contextual research design was used to postulate the coping mechanism of these nurses in a clinical child psychiatric environment. This study comprised psychiatric nurses, and data were collected through focused group interviews. The findings emphasised the importance of clinical support to enhance quality nursing care and adjustment related to the demands on psychiatric nurses to participate fully in the service. Conclusion: Child and mental health services ought to enable nurses to better deal with the service demands and quality of psychiatric health care. An increased understanding of child mental health challenges is needed so that nurses’ roles and functions in child psychiatric practice can be defined. Effective management and support ought to enable psychiatric nurses and health care teams to make decisions that support international development targets.

## 1. Introduction

For the purposes of this article, psychiatric nurses are defined as generalist and non-specialist nurses caring for children in accredited psychiatric institutions. These are psychiatric nurses with basic knowledge of mental health and illness, but without specific knowledge of how they relate to children. However, they have to care for children with mental health challenges. These psychiatric nurses are not always trained sufficiently to work specifically with children with mental illness in their basic training. The South African Nursing Council (SANC) highlights that mental health nurses are professionally equipped to serve the community as specialist mental health care practitioners in mental health establishments. Even though these nurses do not have enough knowledge to care for children diagnosed with mental illness, they are expected to run the services in child psychiatry because of service demands. This justifies questions about the quality of care that patients receive [1].

Child psychiatry is one of the specialisations within psychiatric nursing, and many services consider it an essential component of mental health care for the population [2].

In this area of clinical practice, psychiatric nurses are well placed to support and observe the behaviour of children diagnosed with mental illness. By providing close care and getting to know the child, they have opportunities to recognise and respond to their expressions of mental distress [3].

Caring for mentally ill children is demanding, and nurses need informal and formal support of an emotional and educational nature. Clinical supervision in this environment may lead to increased self-reflection and competence, including enhanced emotional awareness related to transference and countertransference reactions [3].

The literature suggests that whilst there are very few specialist nurses who specialise in caring for children with mental health and emotional challenges specifically, mental illness in children continues to frustrate communities in the North West Province (NWP) of South Africa (SA). Child mental health services in this province are scant and not well defined. There is currently no evidence in the literature on how child psychiatric nursing services are rendered and how these nurses cope with the scant clinical services in this province. These children’s mental illnesses manifest differently compared to those of adults, and the treatment approach is also different. Therefore, other nurses are brought in without the required skills and knowledge to care for these mentally ill children. It should also be noted that children’s behaviour requires an understanding of how they take in and process information [4].

The stress of having to work with new demands for which they are not specifically trained may negatively affect the coping behaviour of psychiatric nursing staff. This affects nurses’ internal being, and negative emotions such as anxiety and nervousness can be evoked. These negative coping strategies increase the negative effects of work stress [5]. Even though research has concluded that psychiatric nurses experience stress in the workplace, little is known in the literature about the coping mechanisms of psychiatric nurses in child psychiatric settings, despite the fact that the prevalence of mental health challenges among children is fairly well understood. These nurses require appropriate coping mechanisms to reduce the stress of work. Researchers describe coping mechanisms as a direct attempt to cope with the stress experienced or what one does to handle emotions that are negative. Psychiatric nurses’ positive well-being and attitude may also contribute to addressing the challenges of the practice environment [6]. Psychiatric nurses play an irreplaceable role in finding ways to cope and improve their work performance.

The main functions of psychiatric nurses are divided into two components: to support people at a reasonable level of functioning who cannot maintain it independently and to facilitate a higher level of functioning in people for whom improvement can be achieved [7]. Boamah [8] stated that registered nurses must demonstrate strong clinical expertise in providing mental health services.

The need for ongoing mental health training for psychiatric health professionals has been identified for over five decades [9]. Training programmes are necessary to guide career advancement avenues, for the recognition of achievement, and to foster collegiality, which in turn increase the knowledge, skills, and attitude of personnel in different clinical institutions, including assisting in meeting work demands and setting goals [10]. Therefore, continuous education and training in the workplace play an essential role in the provision of quality mental health care to patients and promote positive work performance, including job satisfaction.

Nurses constitute the largest group in the health fraternity who care for mental health care users in psychiatric health care services compared to other health professionals [11]. The current basic training of nurses does not provide psychiatric nurses with the appropriate knowledge needed to effectively take care of children with mental health problems. The stress of having to work with new demands for which they are not specifically trained affects the mental health of such nursing staff. In addition, there is a paucity of research that focuses on child mental health nursing in NWP with a specific emphasis on how psychiatric nurses cope in this environment.

It is necessary to understand psychiatric nurses’ insights into ways to cultivate coping mechanisms in the health care environment of NWP. This study aimed to find out what psychiatric nurses felt needed to be done for them to cope with the care of children’s mental health in this environment. This study offers direction as the basis for a set of preliminary coping mechanisms derived from their responses to offer direction for further refinement and development. It is therefore important that psychiatric nurses are protected from experiencing stress, which may lead to burnout.

## 2. Materials and Methods

### 2.1. Study Design

A qualitative and descriptive research approach was used with explorative focused group interviews, as the focus was on exploring and describing the coping mechanisms of psychiatric nurses in a real-life and everyday situation in coping with children in psychiatric clinical environment [12].

A purposive sampling method of five focus groups was used. A total of thirty-two psychiatric nurses participated in five focus groups. The participants consisted of four males and twenty-eight females. The age groups of the participants ranged from 26 to 59. Five of the participants did not disclose their age. Table 1 below gives a summary of the demographic information of the participants. The focus group discussions were conducted from May to July 2022. The first author conducted the focus group interviews which lasted between forty-five minutes and one hour. The below table summarises the demographic information of the participants.

### 2.2. Data Collection

The researcher used face-to-face focus groups to collect data. Five focus groups led to data saturation. The sample of participants consisted of thirty-two black and two white psychiatric nurses according to South African population classifications. Their qualifications ranged from a diploma in comprehensive nursing (*n* = 25), to a bachelor’s degree in nursing (*n* = 5) and a master’s degree in psychiatric nursing (*n* = 4). Both a diploma and a bachelor’s degree are basic qualifications which include psychiatric nursing as a qualification. A master’s degree is a post-basic qualification. The natural setting was used, which included nursing wards and outpatients’ departments in accredited psychiatric institutions. This study adhered to ethical principles governing research, which include respect, autonomy, anonymity, and non-maleficence. The researcher adopted key ethical principles for health research, including beneficence, non-maleficence, autonomy, and justice.

### 2.3. Setting

This study took place in NWP of SA across four districts in accredited psychiatric institutions where child psychiatry services are rendered. Each district has a hospital with 72-hour psychiatric services. Two of the hospitals in two districts are dedicated psychiatric institutions. These hospitals offer psychiatric services to the entire province. NWP is one of the nine provinces in SA. The province has both urban and rural areas but is mainly rural, with mental health care users travelling great distances to access specialised mental health care services. In this province, the psychiatric services are led by the provincial department of health officials. There are district health officials involved in mental health services. Child psychiatric services in this province are scant and not clearly defined. The staffing ratios are unknown in the practical reality.

### 2.4. Data Analysis

The collected data were analysed and Tesch’s method of open coding was utilised for the analysis of the data [12]. An independent coder who is knowledgeable in the field of qualitative studies was used to corroborate the initial coding.

### 2.5. Ethical Considerations

Ethical approval to conduct the study was obtained from the Scientific Committee of the School of Nursing Science and the North-West University Health Research Ethics Committee (HREC Reference Number: NWU-00278-21-S1). Permission to conduct the study was requested and obtained from the North-West Provincial Department of Health, directors of district health services of NWP and the Chief Executive Officers of the accredited mental healthcare institutions examined during this study. Permission was obtained from the participants, and they signed informed consent forms before participating in the study.

## 3. Results

Table 2 below provides an overview of the coping mechanisms of psychiatric nurses in child psychiatric clinical setting, with the main themes identified.

## 4. Discussion

### 4.1. Theme 1: The Demands on Psychiatric Nurses in the Child Psychiatric Environment

Child psychiatric nursing is a unique clinical environment that responds to the growing health challenges associated with mental health illnesses which start in early life. The literature shows that interpersonal engagement accompanied by knowledge, skills, and attitude are important in dealing with mental health challenges, especially with regard to children [13]. Thus, examining the interpersonal process of psychiatric nurses in relation to how they think, sense, and respond to children with mental health challenges as they attempt to engage with them is important. The interpersonal perspective and shared emotional states have in and of themselves a growth-producing capacity for both the child and the psychiatric nurse, because the nurse helps the child to direct attention to him/herself and at the same time respond to life situations [14]. A lack of knowledge about child mental health often leads to frustration and a lack of direction, which may lead to further stress [15]. Working with children can be generally stressful and demanding. This study emphasized how psychiatric nurses in the child psychiatric environment are instrumental in building quality relationships that provide opportunities to satisfy other needs through the following subthemes:

#### 4.1.1. Nature of Placement and Fit for Purpose

Staff participation in the clinical placement process is important and contributes positively to the effective implementation and increased uptake of allocated responsibilities. Nursing staff need to be heard, prepared, and supported because clinical placements promote personal and professional development. Clinical placements of nurses in any institution can be challenging because some of the staff members struggle to integrate into the team and to define their professional self and feel insecure about their competence [16].

When the staff members are engaged in the placement process, they have reasons and opportunities to interact and co-create a fit between the measures relevant to the nursing care and their work context. This may facilitate increased staff commitment and the ability to use the created nursing care measures effectively, fostering high levels of satisfaction and emotional safety [17]. This is supported by the following quote in understanding their environment:

*Focus Group (FG) 2, Participant (P) 3: “So, I am actually confident when dealing with the kids. Actually, I do prefer kids than adults”.*

Knowing and applying psychiatric nurses’ strengths encourages creativity in a workplace. By capitalizing on these nurses’ strengths means recognizing their potential and helping them to apply these strengths in each situation at work. This is a great way to not only satisfy psychiatric nurses’ emotional desires but also build up their self-confidence and work engagement [17]. Psychiatric nurses play a role in developing the self-esteem and social skills of children who are not emotionally stable, including helping them to identify and develop their potential. This is supported by the following quotes in understanding their environment:

*FG1, P2: “Sometimes you have to be playful with the children so that they can feel welcomed, and they must not feel unwanted”.*

and

*FG1, P3: “Others are like, will be having different behaviours, others will be moving around, and others are just quiet. Meaning that if you don’t assess carefully, there might be, or you can’t see that there might be some behavioural problem on this one. Is not like he/she is like that. There has to be a condition that you need to analyse. That’s the children”.*

It is required that psychiatric nurses should be sociable and tolerant, with interpersonal styles and behaviours that reveal understanding, empathy, flexibility, and interest. These nurses need to have the knowledge of psychiatric nursing and childhood because the children carry the knowledge, views, opinions and behaviour fostered during early years of socialisation into their adult years, all of which determine their personal and professional development [18]. If this knowledge is lacking, concerns with regard to the identification of the emotional challenges within the children will be raised.

#### 4.1.2. The Unique Nature of the Context and Environment

Working in a child psychiatric setting is viewed differently from working with adults who are mentally ill. Caregivers’ involvement is important due to the limited capacity of children in decision making. The implementation of child psychiatric services appears to be challenging due to the complex conversations required with already emotionally disturbed children. Multiple perspectives must be considered and balanced rather than in a single decision point [19]. This area of psychiatric nursing is unique to each child. Moreover, the impact of the COVID-19 pandemic became disruptive to this environment. Unprecedented interruptions were experienced during this period. The literature highlights both a psychological and social bearing on the existing limited child-focused environment [20].

Each person involved in the psychiatric care of the children needs to understand their needs and must work towards meeting those needs. In this study, psychiatric nurses explained the uniqueness of this environment through the following quotes:

*FG1, P2: “I think when you work with children you must think. So basically, you must prepare yourself that today, even if I am feeling like this, I must show a positive aspect to children. Because children like playing and stuff. So, you can’t come to a child when you are very serious”.*

and

*FG1, P5: “It works well with children. I have also realised it that with children you must be playful, you must be able to run around. You must be loose. Yes, you must be loose, and you can’t be tight”.*

A psychiatric nurse who works in this environment needs to be professionally mature. One is professionally mature when one can make decisions that reflect decisiveness, self-reliance, independence, and willingness to compromise between personal needs and the demands of the current professional situation [21].

#### 4.1.3. Cohesion and Team Sharing

Team cohesion is unity that helps to foster and develop each team member. A cohesive team can remain united in the pursuit of its objectives. Nurses in child psychiatry feel rewarded in this field if they are empowered with regard to autonomy, diversity, developing potential and multidisciplinary cooperation [22]. Psychiatric nurses need to create an intangible asset in their workplace by speaking to each other and other team members as equal partners. This serves as a collaborative capital that makes their work easier, efficient and gratifying. The following affirms the need for and importance of team sharing:

*FG1, P5: “I think having nurses with different categories and exposure or having different experience and specialities is helpful. Because if there are nurses who are good in medical, they can assist with the medical part of it. Those who are good or having a speciality in surgical, they can help with it. And those that are good with mental health, they can also help”.*

A team of child psychiatric services comprises a large array of professionals, and psychiatric nurses form the cornerstone of this team. It is crucial to understand how this team works together. The working relations can improve if psychiatric nurses and other colleagues trust each other in work situations rather than working independently [23]. While facilitating social connections improves nurses’ morale and engagement at work, promoting positive team sharing can provide these nurses with a healthy work environment and thus improve coping mechanisms [24]. There must be a common objective that each professional has to align themselves with. For a team to succeed, it must acknowledge the special grace immanent in another person’s life [24]. The success of a team depends on each team member’s openness to learning, cultural values, previous problem-solving success, educational skills, and enthusiasm in caring for children. Comprehensive treatment and inter-professional collaboration are important to any health care team. For a team to succeed, a carefully planned operational network of players must be assembled.

#### 4.1.4. Taking Accountability for Own Learning

Education in the workplace is described as in-service training and may influence personal accomplishment. Nursing staff can apply the knowledge gained through educational opportunities created practically in their respective environment. Training programmes have the potential to minimise the effects of stress. Psychiatric nurses’ behaviour and attitude provide a clue regarding children’s real sense because they process information from multiple senses. They must understand the way the child behaves and thinks, and caring for children should be consistent with that [25]. This is confirmed by the following quotations:

*FG1, P1: “Akere (By the way) mostly the training is with adults. We don’t have that info, that insight like how to work with children. Is just that you wena (you) you are trying. But we don’t have more information. So, I read a lot to understand them”.*

*FG3, P1: “I think from my point of view, I would like critical care training especially being the emergency unit. I think we need critical care training because most of the time we get patients from the wards. But then we won’t know exactly how to deal with the physical aspect. So, I would think especially in the emergency unit, I would say critical care”.*

*FG2, P1: “For me outside my formal training, I did… I don’t want to call it informal, but I am extra in terms of how to deal with kids and even go deeper into trauma for kids and play therapy. So, I am actually confident when dealing with the kids. Actually, I do prefer kids than adults. And experience wise, I have also spent some extensive time working in one of the kids’ unit in one of the hospitals in Gauteng. So personally, I would say I am a bit confident in terms of working with children”.*

Self-determination is an underlying theory of empowerment, and it is a guiding attribute in the empowerment process. Psychiatric nurses in this study believe in empowerment, and this involves self-development and self-efficacy, increased understanding of oneself, personal power, and self-management [25,26]. The World Health Organization (WHO) defines attributes of empowerment as including reciprocal interaction, autonomy linked to accountability, shared transfer of power and ultimately greater access to financial and intangible resources such as knowledge and influence [27]. This expansion will assist in career success including recognising, attracting and retaining talented psychiatric nurses [28].

In the early stages of a psychiatric nursing career, it may be advantageous that nurses are placed in more supportive sites which provide clinical supervision. Clinical supervision is thought to serve two primary goals, which include increased competence and the enhancement of caring outcomes. It includes greater self-awareness, self-efficacy, improvement in clinical skills, and greater integration of theory to practice [28]. The clinical supervision and supportive interventions may need to focus on understanding the children’s development. This in turn will enhance feelings of safety and may foster mutual support.

Psychiatric nurses’ career success is based on the individual’s internal environment and character, which involve the individual’s internal interpretations, perspectives, and evaluations of their success as a nurse and in the work environment [28]. The researchers are of the view that the core part of psychiatric nursing should be devoted to managing personal stress and coping skills. Therefore, support systems which include reflective practice must be available and accessible to the psychiatric nurses in this environment to encourage positive outcomes.

### 4.2. Theme 2: The Adjustment Process of Psychiatric Nurses to Participate Fully in Caring for Children in Psychiatric Setting

#### 4.2.1. Management Interventions

As leadership continues to evolve and challenges in healthcare become more complex, there is an increased need for a focus on manager’s engagement, flexibility, collaboration, and collective leadership [29]. The literature suggests productive interpersonal relationships and networking as one of the factors that contribute to success in managerial employees [28].

Management interventions are required so that psychiatric nurses can benefit from them. Nurse managers also function as leaders in any psychiatric institution. They are also responsible for the health of their staff, and the staff are reliant on them for emotional support [30]. These interventions should aim at supporting psychiatric nurses to be productive in a work environment and improving their skills. There is also a need for constant information and communication about changes in nursing care including about the opportunities and challenges for engaging with staff [31].

The following quote elaborates the role of managers:

*FG1, P9: “Also having more training for nurses on child mental health and adult psyche. And the support and understanding of management. Because sometimes even if they can be trained you find that the managers don’t have skills and understanding. It will be just a fruitless exercise”.*

Nurse managers are responsible for decisions regarding both staff and patients, planning nursing care and the oversight of nurses who provide this care. They also support the staff through time scheduling and providing resources including emotional support. To achieve these, there should be an increased need for continual and constant communication [32].

The nurse manager has a defining role which is important to the achievement of workplace outcomes [33]. Psychiatric nurse managers and management may play a pivotal role in establishing and maintaining supportive conversations. Nurse managers are important catalysts for stimulating conversations between co-workers in the workplace and fostering opportunities to create a collaborative capital [34]. In this province, managers and nursing supervisors need to be knowledgeable and capable regarding child psychiatry for them to offer appropriate support.

#### 4.2.2. Continuous Professional Development

Staff development refers to a well-planned, comprehensive system of continuing professional growth activities, which are carried out over a period to achieve specific goals and objectives. Staff development provides psychiatric nurses with the knowledge, skills and attitude needed to perform efficiently. It encourages individuals to realise the need to improve their own performance. Staff development serves to enhance support, and helps nurses to consolidate their competencies [23].

These quotations support the need for development in this environment:

*FG1, P1: “I would like to specialize because I need training and development on mental needs especially with children. We don’t have that info, that insight like how to work with children. Is just that you wena (meaning you) are trying. But we don’t have more information”.*

and

*FG3, P1: “I think from my point of view, I would like critical care training especially being the emergency unit. I think we need critical care training because most of the time we get patients from the wards. But then we won’t know exactly how to deal with the physical aspect. So, I would think especially in the emergency unit, I would say critical care”.*

There is a clear need for an increased understanding of psychiatric practices amongst children in mental health institutions. There is also a need for continuing professional development to ensure psychiatric nurses possess the skills and confidence to enhance their scope in providing quality care to children. The availability of training opportunities and postgraduate programs in child psychiatry may contribute positively to the development of these professionals [23]. Education and professional development sessions must be conducted in the unit for every nurse engaged in caring for children with mental illnesses [24].

#### 4.2.3. Development of All Categories of Staff through In-Service Training

In-service training entails education of employees while they are doing the job. It suggests updating, training, educating and informing the person about the present requirement of the job. In-service education programmes are usually directed towards bringing employees up to date about new developments in treatment and care. The aim of in-service education is to fill the gaps in learning or remedy deficiencies in the skills and knowledge of employees. There is a need for training programs of nurses in child psychiatric settings in order for them to gain useful knowledge [28]. This is affirmed by the narrative below:

*FG1, P1: “We need training and development on mental needs especially with children”.*

In the study, it was clear that the psychiatric nurses need to be trained for them to function more efficiently. The following quote affirms this need:

*FG1, P9: “So, managers need to be trained. Yes, to have an understanding on mental health and child psychiatry. So that they can know what to support, how to support and when to support”.*

Staff development is a key to quality health care. Staff development has been a continuous concern of all health care professionals. It ensures the optimal utilisation of staff in any working environment. Every staff member needs to be developed according to their own needs and the type of work that they are doing [26].

A professionally competent psychiatric nurse displays several professional attributes required for their practice [35]. These attributes include knowledge and experience which remains within people. Researchers also found that the five most frequently observed professional behavioural attributes in psychiatric nursing are empathy, enthusiasm, being personable, having a positive attitude, and responsibility [36].

Therefore, nurses in the child psychiatric environment must do everything in their power to update their knowledge and obtain new insights in child psychiatry by reading reports and attending and participating in workshops with a view to acquire new skills.

With the current dispensation of psychiatric nurses not being produced annually as before, it is imperative to ensure that the entire training program caters for the clinical needs of these professionals. For an employee to perform satisfactorily, the skills, abilities, and attitude for performing the job must match the job’s requirements [37]. A mismatch may lead to poor performance, absenteeism, turnover and other work-related problems. Continuous development to enhance focus, support and ongoing investment in the mental health of psychiatric nurses in a workplace is needed.

#### 4.2.4. Relevant Child Psychiatry Nursing Curriculum

The primary knowledge base of psychiatric nursing includes mental health and mental illness. It includes the foundational application of sensitive, situational attention and observation, including emotional presence. However, the basic training of nurses lacks this aspect. The establishment of a teaching model that effectively integrates child mental health in the basic training of a nurse is currently a challenge in SA [38]. Knowledge, skills, attitude and understanding remain fundamental in the training of a psychiatric nurse. The training of psychiatric nurses should address the service needs and demands. The literature highlights that the ability to identify mental health problems in children is as important as intervening early. The following quote affirms the need for relevance in nursing education and practice:

*FG1, Participant 8* *: “For me my basic training, is not helpful with basics of working with children. Because the time I was doing mental health training it was adult psychiatry. Nothing really about the child was done. So, it was very difficult to deal and understand the children because children are playing, they are running around and sometimes you don’t even know or aware that this child is having a problem. Because the child is a child. I don’t think what we have done, or we would have been aware of a child mental health if it was not for practice”.*

It is extremely difficult when the nurse does not have the appropriate knowledge and skills to care for a child with mental illness and more difficult when emerging emotional problems are complex. In this study, psychiatric nurses exhibited limited or no exposure to child psychiatry during their basic training, and this is supported by the following quote:

*FG1, Participant 6: “Mostly the training is with adults. We don’t have that info, that insight like how to work with children”.*

The curriculum for psychiatric nursing should value children with mental illness as critical clients in the health care system. It should focus on the foundation for professional competence that promotes recovery within a therapeutic environment and relationship. Curriculum development and change are the consequences of changes which take place in people and the environment they live in [39]. Improved child psychiatric nursing training and programmes need to be established in nursing education institutions to address the service demands, knowledge and skills of psychiatric nurses. An increased focus on theoretical and clinical experiential learning in child psychiatric nursing is likely to produce more positive attitudes towards the nurses and towards children in this environment [40].

Therefore, psychiatric nursing students need to be well prepared to meet the needs of children with mental illness. These nurses need to provide safe and effective care to children experiencing mental health challenges. For the clinical practice environment to expect psychiatric nurses to provide nursing care to children in an area that they have little or no knowledge is essentially a contravention of their scope of practice and basic human rights [36].

It was established almost two decades ago that, even though psychiatric nurses play an important role in the identification of mental illnesses and the referral system of the health department, they usually lack the necessary comprehensive assessment needed to identify the signs of mental health challenges and disorders in children [41]. Therefore, the implementation of an effective training program and its impact are key factors in ensuring that the psychiatric nurses put theory into practice. It is an urgent priority to train psychiatric nurses in child and adolescent psychiatry and to develop systems in which their expertise can be utilised to the maximum [8].

#### 4.2.5. Flexible Time Shifts

Flexibility is regarded as offering choices in the practice environment to meet the needs of individual practitioners. Flexible time shifts allow psychiatric nurses to take some time out to recuperate. In this study, the predominant nursing tasks and responsibilities of psychiatric nurses appeared insufficient to respond to patients’ fluctuating needs. In addition, shorter working days produced an undesirably excessive level of flexibility among them. Where possible, psychiatric nurses should be given more control over their shifts, which will allow them to perform their job in the best and most effective way possible. This is likely to have a positive influence on burnout. The following affirmations confirm the need for flexibility.

*FG5, P2: “Even the clinic to run Monday to Friday without limitations till 4pm”.*

*FG5, P1: “I think if there was a clinic that is running on the daily basis, that thing would be possible but because of limited time for them to be seen by the psychiatrist, everything is fast fast fast”.*

*FG2, P3: “For the system to work better is for the clinic to run every day. And for the ward to open up and for the MDT team meetings to take place. All the members to take part in that. In that we will know that our patients are getting proper care”.*

The traditional personnel approaches that were conceived in cultures emphasize command and control. The new approaches include and are described by employee commitment, cooperation and communication. Today, it is important to develop the capacity for flexibility and this is an important component of any company’s corporate human resource strategy [40]. It is no longer enough to provide treatment and rehabilitation to nurses who are already experiencing stress and emotional exhaustion. One must rather prevent and protect these psychiatric nurses [9].

### 4.3. Theme 3: The Need for Outreach Programs

Outreach programs in this study involve providing health education, health support, and other projects for welfare. Resources must be used fulfil these goals. An outreach program aims to assist and support those who are deprived of certain services and rights. In this study, the participants outlined the need through the below subthemes.

#### 4.3.1. Creating Social Awareness of Child Psychiatry

Social awareness enhances consciousness of the difficulties around mental health in children and the hardships encountered by children exhibiting emotional challenges. It also fosters understanding of Indigenous culture and its impact on children’s mental health. Social awareness also emphasises the importance of cultural safety informed by awareness and understanding of social, cultural and historical factors that can impact the mental health and treatment of children [42].

Research has shown a positive relationship between child mental health and an increase in self-esteem, as these two will demonstrate positive social behaviours and characteristics such as assertiveness, social abilities, and adaptability in various events and conditions [34]. The literature relating to child mental health focuses largely on changes in the knowledge base and attitude, but falls short of considering the primary focus of health education, its impact upon practice and how this ultimately benefits children with mental health challenges. Psychiatric nurses caring for children in these communities have affirmed this through the following quotes:

*FG1, P8: “Even if you can understand how to work with children, children are coming from different homes and from different families. Where they don’t really understand when the child is having a problem. So maybe strengthening mental health services at the community also needs to be attended to”.*

and

*FG1, P4: “I think it would be best if we can have a team or nurses that would go for outreach programs”.*

Rural and underserviced communities are particularly impacted by mental health challenges, with resulting intergenerational effects on social and emotional wellbeing. The literature indicates that the mental health concerns of children impacted by the coronavirus pandemic have not been adequately addressed. There can be challenges faced by psychiatric nurses when caring for children with mental health problems in these communities. There is a need to focus on the promotion of the social and emotional wellbeing of children and not simply the treatment of symptoms for meaningful practice and culturally appropriate child mental health care services [38]. The following quote confirms this:

*FG1, P8: “I think even the society itself needs to have an understanding of children”.*

The public awareness of mental health challenges among children remains limited amongst NWP communities. These communities need to be better prepared to understand behavioural problems amongst children. These include non-compliance with social norms including values and rules, e.g., making eye contact, running away from home, truanting from school, being cruel to animals and bullying others. The community members need to know that when children have such ways of speaking, behaving, and playing which represent relationship problems and differ from the other children’s expectations, they tend to hurt and disrupt the internal peace of others [43].

There is also a need for psychiatric nurses to partner with communities to find out what will work to improve the social and emotional wellbeing of children as service users and to articulate this with the evidence base for practice.

#### 4.3.2. Discussion and Support Groups

Discussion happens when team members engage in sharing experiences and encounters as a team. This discussion builds the team and emerges from a clear vision of what the team wants to achieve. The discussion groups form part of the support systems for psychiatric nurses in a workplace. Supportive workplace conversations allow for the provision of comfort and expression of support and reassurance to colleagues in distressing circumstances in a highly demanding work environment. Supportive conversations restore confidence and can assist with conflict resolution [23].

Social support networks should be encouraged amongst psychiatric nurses as this provides support against job stressors and are bound to ultimately reduce burnout [19]. Teamwork in the form of multi-professional collaboration, teaching and interdependence must be strengthened. The co-operation between different departmental services, for example social services and child psychiatry, inter-professional sharing and training can be considered valuable options for psychiatric nurses. This would enhance and empower psychiatric nurses in the process and benefit the collaborative work practice in this area [44].

## 5. Limitations of the Study

The findings from this study should be viewed within the context of its limitations. This is a small-scale study which followed a qualitative research approach, and the findings cannot be generalised because each context is unique. A similar study can be replicated in other provinces. Future research with larger sample sizes will allow for a more robust discussion.

## 6. Conclusions

Child psychiatry remains outside of the priorities and under resourced in North West Province. At the same time, psychiatric nurses are faced with challenges in their daily practice which require them to make rational and critical clinical decisions including clinical judgments that are logical to the child who is emotionally not well [44]. However, efficiency and equity in this area of service provision may be lacking due to insufficient provincial coordination. Additionally, the efficacy of services is not being evaluated, leaving behind the opportunity for quality improvement. Effective management and support must enable psychiatric nurses and health care teams to make decisions which support the international development targets.

Mental disorders are part of the chronic diseases of children and adolescents. Prevention of these disorders offers an opportunity to reduce the public health burden of mental illness by mitigating risks and promoting mental health before mental conditions are present. Promotion of early childhood mental health may have an impact on overall health, disease, and human capital across the entire life of a person [42]. However, it is also important to know when to act based on the evidence which is propelling us forward like research. Public health care leaders should use such evidence to improve the lives of communities. Pirson [45] confirmed that the notion of dignity as that which has intrinsic value has been neglected in health care, despite its societal importance. The identified gap in human resources must be attended to urgently.

This study highlights the need for training in child psychiatry. A stand-alone child psychiatric mental health nursing course must be considered with dedicated child psychiatric clinical hours. It considers a training package provided to all frontline care staff in psychiatric institutions, general hospitals, community health centres and clinics where children with mental health challenges first present and servicing young people between the ages of three and eighteen with mental and emotional problems. This study is expected to guide various stakeholders in both practice and higher education to identify challenges and develop strategies for creating a better life for all.

Children in mental health settings need the highest possible level of care offered to them. It is required of psychiatric nurses to demonstrate the art of nurturing by applying professional competencies and positive emotions. Clinical support to enhance the mental health of nurses in child psychiatric environment is important. There is a need for an emphasis on caring and support for psychiatric nurses offering mental health services to children. Therefore, the ethics of care in this unique environment need to be acknowledged. Further research is needed to explore these themes further, to refine and develop guidelines and the development of programmes for offering the necessary support and empowerment to psychiatric nurses in this clinical environment.

## Figures and Tables

**Table 1:** Demographic information of participants.

Focus Group 1	Age	Gender	Qualifications
P1	58	F	Diploma in general community, psychiatry and midwifery nursing
P3	52	M	Diploma in general community, psychiatry and midwifery nursing
P2	46	F	Bachelor’s degree in nursing
P4	47	F	Diploma in general community, psychiatry and midwifery nursing
P5	26	M	Bachelor’s degree in nursing
P6	42	F	Diploma in general community, psychiatry and midwifery nursing
Focus Group 2	Age	Gender	Qualification
P1	42	F	Diploma in general community, psychiatry and midwifery nursing
P3	46	F	Diploma in general community, psychiatry and midwifery nursing
P2	-	F	Diploma in nursing
P4	-	F	Diploma in general community, psychiatry and midwifery nursing
P5	36	M	Diploma in general community, psychiatry and midwifery nursing
P6	-	F	Master’s in psychiatric nursing
P7	-	F	Diploma in general community, psychiatry and midwifery nursing
P8	53	F	Master’s in psychiatric nursing
P9	46	F	Diploma in general community, psychiatry and midwifery nursing
Focus Group 3	Age	Gender	Qualifications
P1	38	F	Master’s in psychiatric nursing
P3	59	F	Master’s in psychiatric nursing
P2	34	F	Bachelor’s degree in nursing
P4	52	F	Diploma in general community, psychiatry and midwifery nursing
Focus Group 4	Age	Gender	Qualifications
P1	54	F	Master’s in child psychiatry
P3	51	M	Bachelor’s degree in nursing
P4	34	F	Diploma in general community, psychiatry and midwifery nursing
P5	36	F	Diploma in general community, psychiatry and midwifery nursing
P6	38	F	Diploma in general community, psychiatry and midwifery nursing
P7	42	F	Diploma in general community, psychiatry and midwifery nursing
Focus Group 5	Age	Gender	Qualifications
P1	45	F	Diploma in nursing
P3	36	F	Diploma in nursing
P4	41	F	Diploma in general community, psychiatry and midwifery nursing
P5	43	F	Diploma in general community, psychiatry and midwifery nursing
P6	-	F	Diploma in nursing

**Table 2:** Summary of themes and subthemes.

Themes	Subthemes
Theme 1: The demands on psychiatric nurses in the child psychiatric environment	Nature of placement and fit for purpose. The unique nature of the context and environment Cohesion and team sharing Taking accountability for own learning
Theme 2: The adjustment of psychiatric nurses to participate fully in caring for children in the psychiatric setting	Management interventions Continuous professional development Relevant child psychiatric curriculum Flexible time shift
Theme 3: The need for outreach programs	Creating awareness of child psychiatry Discussion and support groups

## Data Availability

The data presented in this study are available on request from the corresponding author. The data are not publicly available due to ethical reasons.
